# Growth promotion on maize and whole-genome sequence analysis of *Bacillus velezensis* D103

**DOI:** 10.1128/spectrum.01147-24

**Published:** 2024-11-07

**Authors:** Yating Zhang, Ning Zhang, Xinyue Bi, Tong Bi, Faryal Babar Baloch, Jianjia Miao, Nan Zeng, Bingxue Li, Yingfeng An

**Affiliations:** 1 College of Bioscience and Biotechnology, Shenyang Agricultural University, Shenyang, China; 2 College of Land and Environment, Shenyang Agricultural University, Shenyang, China; Tianjin University, Tianjin, China

**Keywords:** *Bacillus velezensis*, PGPR, genome sequencing, maize, rhizosphere

## Abstract

Root-associated microorganisms, particularly plant growth-promoting rhizobacteria (PGPR) from the *Bacillus* genus, play a crucial role in enhancing crop yield and health. In this study, a *Bacillus* strain was isolated from the rhizosphere soil of maize and identified as *Bacillus velezensis* D103. The primary objective of this research was to evaluate the potential of D103 as a PGPR. Laboratory tests demonstrated that D103 is capable of nitrogen fixation, inorganic phosphorus solubilization, potassium solubilization, and the synthesis of indole-3-acetic acid, ammonia, siderophores, amylase, protease, cellulase, β-1,3-glucanase, and 1-aminocyclopropane-1-carboxylate deaminase. Additionally, D103 exhibited swimming and swarming motility, biofilm formation, and an antagonistic activity against pathogenic fungi. Genome mining identified genes associated with growth promotion and biocontrol activities. In a hydroponics experiment, maize plants treated with a D103 suspension at a cell density of 10^3^ CFU·mL^−1^ resulted in the most pronounced showed significant growth stimulation, with shoot length and total root length increasing by 43% and 148%, respectively. These results support the potential of D103 as an effective PGPR for promoting maize crop growth.

**IMPORTANCE:**

In this study, we assessed the capacity of D103 to promote plant growth and examined the effects of hydroponic experiments inoculated with this strain on the growth of maize seedlings. We sequenced and analyzed the complete genome of D103, identifying several genes and gene clusters associated with plant growth promotion and resistance to pathogenic fungi, thus revealing the plant growth-promoting mechanisms of this strain. The isolation and characterization of new strains with beneficial traits are essential for expanding microbial resources available for biofertilizer production. Collectively, these findings highlight the promising potential of *Bacillus velezensis* D103 as a biofertilizer for agricultural applications.

## INTRODUCTION

Modern agricultural practices have significantly enhanced crop yields over the past few decades, primarily through the extensive application of fertilizers and chemical pest control methods (1). However, the reliance on chemical fertilizers and manure to enhance soil fertility and crop productivity has adversely effected complex biogeochemical cycling processes (2). In response to these concerns, scientists and farmers worldwide are adopting organic farming practices, which incorporate traditional agricultural techniques and innovative technologies to replace chemical fertilizers and hazardous pesticides with organic fertilizers and biological control agents (3). Consequently, there is a growing focus on developing novel strategies for organic technologies and exploring their integration with conventional agrochemicals to promote a more sustainable and environmentally friendly agricultural system.

Rhizosphere bacteria that facilitate plant growth by providing essential nutrients and regulating plant processes are recognized as plant growth-promoting rhizobacteria (PGPR) (4). PGPR are considered a promising alternative to conventional fertilizer due to their environmentally friendly nature. These beneficial bacteria colonize the root surface or the rhizosphere and enhance plant growth through direct mechanisms such as biological nitrogen fixation (5), mineral solubilization, and production of various phytohormones (6). In addition, PGPR influence plant health through indirect mechanisms, including the production of siderophores (7), 1-aminocyclopropane-1-carboxylate (ACC) deaminase activity (8), volatile organic compound (VOCs) (9), antifungal activity, and induced systemic resistance (ISR) (10). Numerous genera of PGPR have been extensively studied and applied globally to evaluate their plant growth-promoting (PGP) potential, including *Agrobacterium* (8), *Bacillus* (11), *Burkholderia* (12), and *Pseudomonas* (11). These PGPR have demonstrated significant value in sustainable crop production.

Numerous *Bacillus* spp. have been recognized as PGPR and are commercially employed as biofertilizers due to their ability to produce resistant endospores, suppress pathogens, and promote plant growth (13). Among these, *Bacillus amyloliquefaciens*, *Bacillus licheniformis*, and *Bacillus subtilis* are the most extensively utilized species (14). In 1999, *Bacillus velezensis* (CR-502^T^) was originally isolated from environmental samples collected from the estuary of the Vélez River, Spain (15). Recently, several strains of *B. velezensis* have gained significant interest for their PGP capabilities, enhancing yield and improving product quality in both greenhouse experiments and field trials (16, 17). *B. velezensis* FZB42 has been formulated into a commercially available inoculant, RhizoVital, to control various soilborne diseases and promote plant growth (18). Genome sequencing strategies have facilitated the investigation of plant growth-promoting genes and secondary metabolite gene clusters in *Bacillus* strains, aiding in the identification of potential PGPR or biocontrol agents (19). Consequently, genetic studies and whole-genome comparisons are highly effective tools for understanding the biological characteristics of PGPR strains.

This study aimed to investigate the PGP properties of *B. velezensis* D103, isolated from the maize rhizosphere. Specifically, the study evaluated (i) potential nutritional contributions, including mineral solubilization and siderophore production; (ii) biochemical and enzymatic functions; (iii) antagonistic effects against fungal pathogens; (iv) genomic analysis and comparative genomics to elucidate the genetic basis of PGP activities and phytopathogen antagonism; and (v) the impact on maize growth.

## RESULTS

### Identification of strain D103

**Fig 1:**
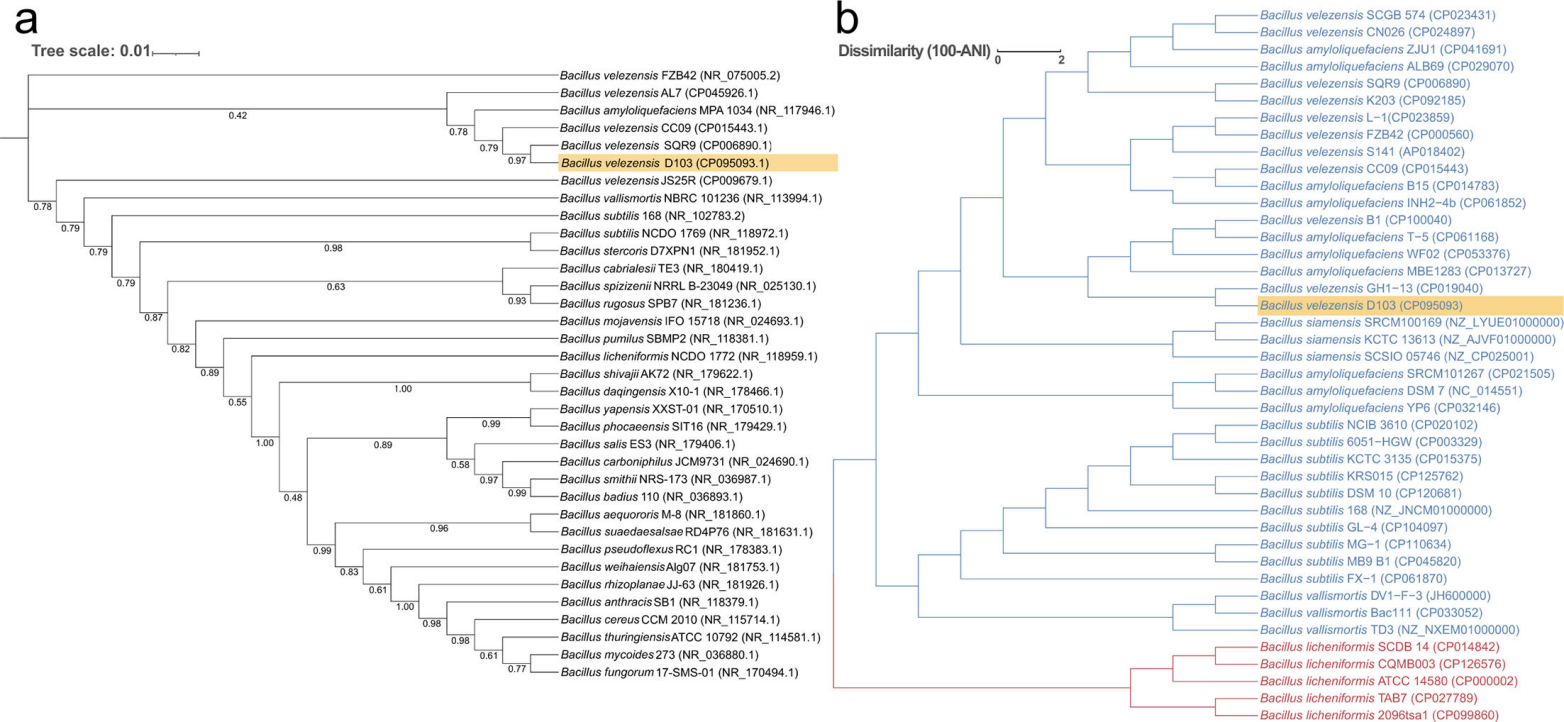
Taxonomic classification of *Bacillus* sp. downloaded from the National Center for Biotechnology Information Reference Sequence Database. (a)Phylogenetic tree depicting the relationships among *Bacillus velezensis* D103 and various other *Bacillus* strains based on 16S rRNA gene sequences. Phylogenetic relationships were determined using a maximum parsimony method, with support values derived from 1,000 replicates. (b)Pairwise average nucleotide identity (ANI) results between 42 *Bacillus* genomes. The similarity among all genomes is represented by a dendrogram using average linkage hierarchical clustering with tree heights corresponding to ANI similarity. Blue branches indicate most of the *Bacillus* genomes, while red branches represent different genomes of *Bacillus licheniformis* sp.

The morphological examination identified that D103 is a Gram-positive strain capable of spore production. Molecular identification was performed through sequencing of the 16S ribosomal RNA (rRNA) gene (1,547 bp). Basic Local Alignment Search Tool analysis and phylogenetic tree results confirmed that D103 was *Bacillus velezensis* (Fig. 1a). Average nucleotide identity (ANI) results showed that D103 shared more than 98.5% homology with *Bacillus velezensis* GH1-13, *Bacillus amyloliquefaciens* WF02, *Bacillus amyloliquefaciens* MBE1283, *Bacillus velezensis* B1, and *Bacillus amyloliquefaciens* T-5, with *Bacillus velezensis* GH1-13 showing the highest level of similarity (Fig. 1b; Fig. S1).

### Characterization with beneficial traits of strain D103

**Fig 2:**
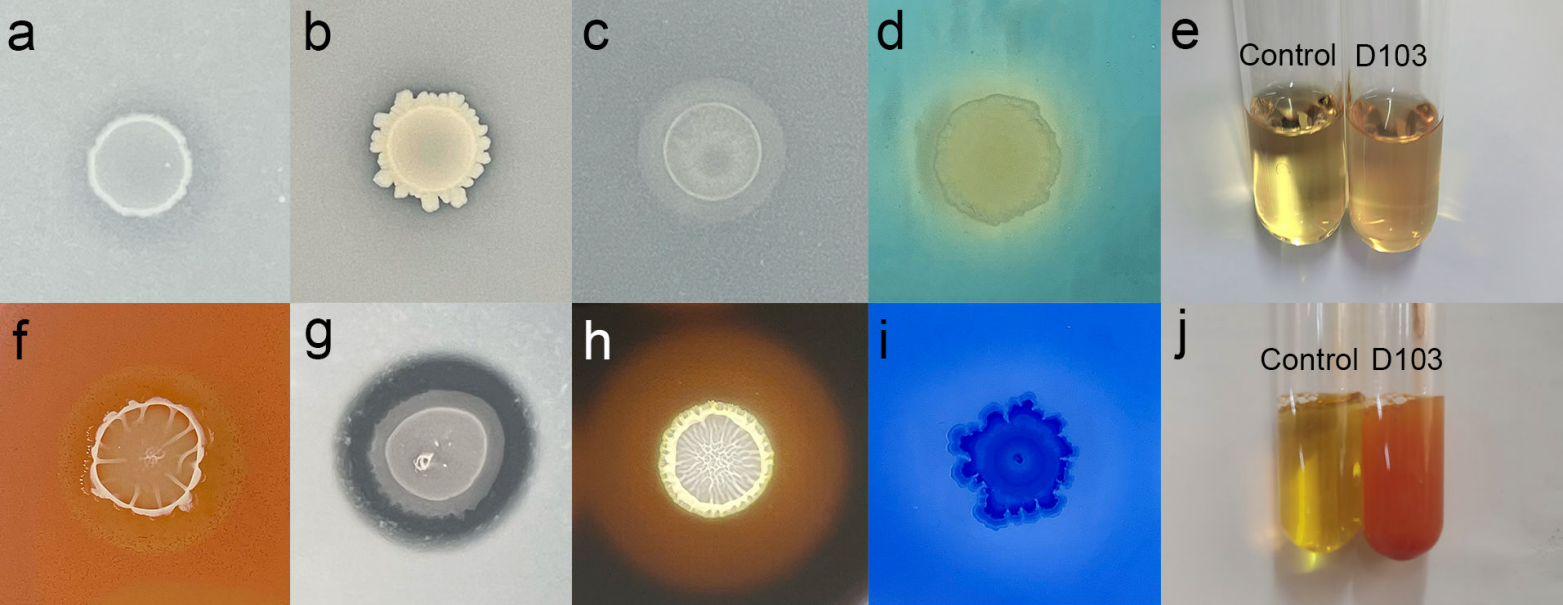
Experimental assessments of plant growth-promoting and hydrolytic enzyme production properties in strain D103. (a) Nitrogen fixation, (b) inorganic phosphorus solubilization, (c) potassium solubilization, (d) siderophore production, (e) IAA production, (f) cellulase production, (g) protease production, (h) amylase production, (i) β-1,3-glucanase production, and (j) ammonia production.

To understand the mechanisms behind the plant growth-promoting effects of strain D103, we evaluated its capacity for nitrogen fixation, inorganic phosphate solubilization, potassium solubilization, and indole-3-acetic acid (IAA) production. Strain D103 demonstrated nitrogen-fixing ability through its growth on nitrogen-free media (Fig. 2a). Nitrogenase activity, measured using the acetylene reduction assay, was 102.49 nmol ethylene·ml^−1^·h^−1^ (Table 1). Additionally, incubation with Pikovaskaia inorganic phosphate medium and Aleksandrov potassium-solubilizing medium resulted in clear zones around D103 colonies, indicating its proficiency in solubilizing inorganic phosphorus and potassium (Fig. 2b and c). Quantitative assessments revealed that D103 solubilized inorganic phosphate at a concentration of 68.67 mg·L^−1^ (Table 1). Furthermore, strain D103 produced IAA, as demonstrated by the Salkowski test (Fig. 2e), with ultra-performance liquid chromatography (UPLC) analysis showing a production level of 31.60 mg·L^−1^ (Fig. S2; Table 1).

**TABLE 1:** Plant growth-promoting factors and hydrolytic enzymes produced by *Bacillus velezensis* D103 at different concentrations

Characteristics	Activity rate
Nitrogenase activity	102.49 ± 3.40 nmol ethylene·ml^−1^·h^−1^
Inorganic phosphate solubilization	68.67 ± 4.19 mg·L^−1^
IAA production	31.60 ± 2.15 mg·L^−1^
Biofilm production	1.121 ± 0.094
Polysaccharides of EPS^*a*^	58.73 ± 3.25 mg·L^−1^
Proteins of EPS	32.05 ± 0.66 mg·L^−1^
Nucleic acids of EPS	8.07 ± 0.87 mg·L^−1^
Amylase activity	6304.55 ± 354.78 U·L^−1^
Cellulase activity	254.89 ± 21.84 U·mL^−1^
Protease activity	545.52 ± 55.63 U·L^−1^
ACC deaminase activity	32.55 ± 0.92 IU·mL^−1^
β−1,3-Glucanase activity	150.83 ± 4.22 U·L^−1^

^*a*^ EPS, extracellular polymeric substance.

### Strain D103 produces ammonia and siderophores, possesses surface motility, and forms biofilms

Strain D103 produced ammonia, as indicated by the brown coloration of the culture liquid following the addition of Nessler’s reagent, confirming a positive result (Fig. 2j). The appearance of a yellow color around D103 colonies on Chrome Azurol S (CAS) agar medium further demonstrated the strain’s ability to produce siderophores (Fig. 2d). Motility assessments were conducted on media with varying agar concentrations (0.3% for swimming and 0.7% for swarming). The maximum colony diameter of D103 was observed after 12 h on swimming media and after 18 h on swarming media (Fig. S3). Additionally, strain D103 formed biofilm, as determined by the crystal violet staining method (Table 1). Quantitative analysis of the extracellular polymeric substance (EPS) extracts revealed that the EPS of D103 comprised 58.73 mg·L^−1^ of polysaccharides, 32.05 mg·L^−1^ of proteins, and 8.07 mg·L^−1^ of nucleic acids (Table 1).

### *In vitro* hydrolytic activity of strain D103

In the *in vitro* analysis (Fig. 2f through i; Table 1), strain D103 demonstrated hydrolytic activity, producing cellulase, protease, amylase, β-1,3-glucanase, and ACC deaminase. The enzyme activities measured for D103 were 254.89 U·mL^−1^, 545.52 U·L^−1^, 6304.55 U·L^−1^, 150.83 U·L^−1^ and 32.55 IU·mL^−1^, respectively.

### Antagonistic activity against fungal pathogens

**Fig 3:**
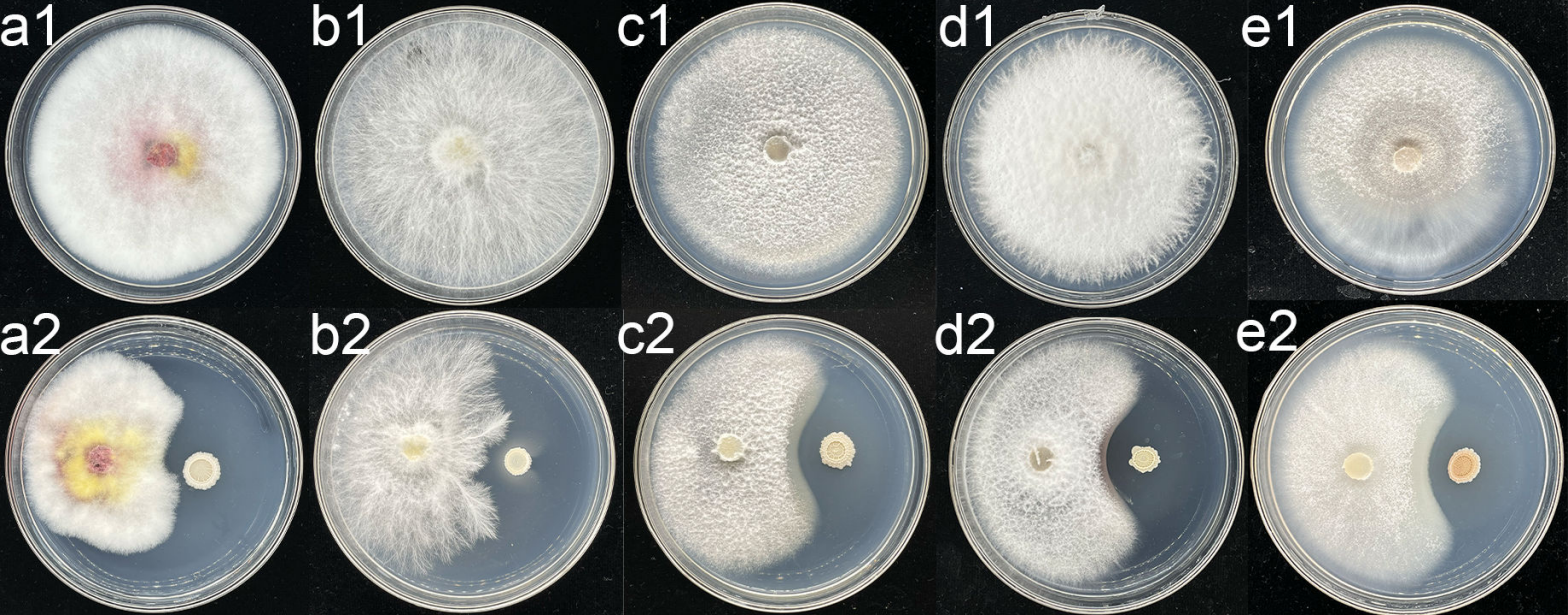
Antifungal activity of strain D103 against various plant pathogenic fungi. (a1 through e1) Placement of a 1-cm agar plug in the center of each potato dextrose agar plate. (a2 through e2) Incubation of strain D103 3.5 cm away from the fungal plugs. Pathogenic fungi included (a) *Fusarium graminearum*, (b) *Athelia rolfsii*, (c)*Fusarium thapsinum*, (d) *Gibberella fujikuroi*, and (e) *Gibberella moniliformis*.

*In vitro* analyses involving five plant pathogenic fungi, demonstrated that strain D103 effectively inhibited the growth of *Fusarium graminearum*, *Athelia rolfsii*, *Fusarium thapsinum*, *Gibberella fujikuroi*, and *Gibberella moniliformis* (Fig. 3).

### Genomic characterization of strain D103

**Fig 4:**
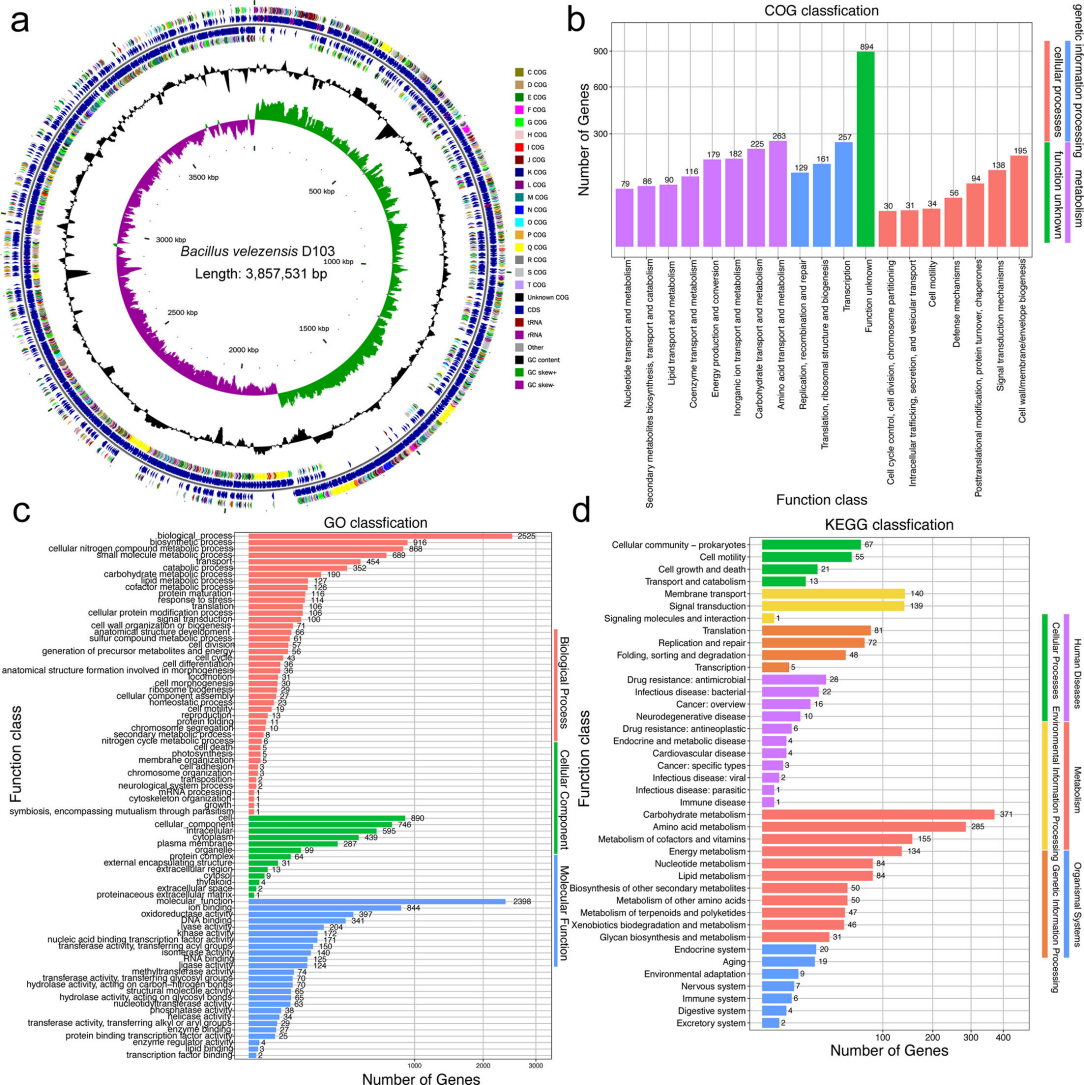
Database annotation for *B. velezensis* D103. (a) A circular genome map is presented, showing the scale; GC skew; GC content; COG classifications for coding DNA sequences (CDS); and the specific positions of CDS, transfer RNA (tRNA), and ribosomal RNA (rRNA) on the genome. This map offers a comprehensive overview of the genomic structure. (b) COG database annotation, (c) GO database annotation, (d) KEGG database annotation.

The genomic characterization of strain D103 is shown in Fig. 4a, highlighting key features. The genome consists of a circular chromosome measuring 3,857,531 bp, with an average guanine-cytosine (GC) percentage in DNA (GC content) of 46.7%. It contains 3,884 protein-coding genes, 27 rRNA genes, and 86 transfer RNA (tRNA) genes. Functional analysis of the genome sequences was performed using the Cluster of Orthologous Groups of Proteins (COG), Gene Ontology (GO), and Kyoto Encyclopedia of Gene and Genomes (KEGG) databases. The assignments of the genes in these databases are presented in Table 2; Fig. 4.

**TABLE 2:** Functional categorization of the genome of strain D103 in the COG database

COG categories	Category function	ORF^*a*^ number
A	RNA processing and modification	0
B	Chromatin structure and dynamics	0
C	Energy production and conversion	179
D	Cell cycle control, cell division, and chromosome partitioning	30
E	Amino acid transport and metabolism	263
F	Nucleotide transport and metabolism	79
G	Carbohydrate transport and metabolism	225
H	Coenzyme transport and metabolism	116
I	Lipid transport and metabolism	90
J	Translation, ribosomal structure, and biogenesis	161
K	Transcription	257
L	Replication, recombination, and repair	129
M	Cell wall/membrane/envelope biogenesis	195
N	Cell motility	34
O	Posttranslational modification, protein turnover, and chaperones	94
P	Inorganic ion transport and metabolism	182
Q	Secondary metabolites biosynthesis, transport, and catabolism	86
R	General function prediction only	0
S	Function unknown	894
T	Signal transduction mechanisms	138
U	Intracellular trafficking, secretion, and vesicular transport	31
V	Defense mechanisms	56
W	Extracellular structures	0
Y	Nuclear structure	0
Z	Cytoskeleton	0

^*a*^ ORF, open reading frame.

Among the 3,884 genes present in the D103 genome, 3,239 genes were categorized into 19 categories of the COG database, while 645 genes remained uncategorized (Fig. 4b; Table 2). The largest number of genes was classified into the category of functionally unknown proteins (S), comprising 23.02% with 894 genes. This was followed by amino acid transport and metabolism (E) with 263 genes (6.78%), transcription (K) with 257 genes (6.62%), carbohydrate transport and metabolism (G) with 225 genes (5.79%), cell wall/membrane/envelope biogenesis (M) with 195 genes (5.02%), and energy production and conversion (C) with 179 genes (4.61%).

The GO database identified 2,678 genes in D103. Among these, 1,364 genes were associated with molecular functions related to binding; 19 genes were associated with cell motility; and 5 genes were associated with locomotion. These functions are associated with the strain’s ability to form biofilms and colonize plant tissues (20) (Fig. 4c). Using the KEGG database, we assessed the potential involvement of D103 genes in biological pathways, resulting in the classification of 2,152 genes. The KEGG pathway analysis categorized these genes into 40 functional pathways. The most represented pathways included carbohydrate metabolism (256 genes), amino acid metabolism (371 genes), amino acid metabolism (285 genes), metabolism of cofactors and vitamins (155 genes), membrane transport (140), and signal transduction (139 genes) (Fig. 4d).

### Comparative genetic characterization among *Bacillus* spp.

Comparative genomic characterization was conducted among *Bacillus* spp., including reference strains *Bacillus velezensis* FZB42, *Bacillus velezensis* EN01, *Bacillus subtilis* 168, and *Bacillus amyloliquefaciens* X030. Genome sequences were obtained from the National Center for Biotechnology Information (NCBI) database and compared with strain D103, as presented in Table 3. These five strains demonstrated overall similarity in genome size and the number of coding genes. However, differences in the number of genomic islands and prophages were observed, potentially contributing to variations in their genetic profiles. Genomic islands and prophages serve as mobile genetic elements facilitating horizontal gene transfer and play a role in bacterial adaptation and evolution (21).

**TABLE 3:** Comparative genomic analysis of *B. velezensis* D103, *B. velezensis* FZB42, *B. velezensis* EN01, *B. subtilis* 168, and *B. amyloliquefaciens* X030

General genomic characterization	*B. velezensis* D103	*B. velezensis* FZB42	*B. velezensis* EN01	*B. subtilis* 168	*B. amyloliquefaciens* X030
NCBI accession	CP095093	NC_009725.2	NZ_CP053377.1	NZ_CP010052.1	NZ_CP040672.1
Size (bp)	3,857,531	3,918,596	4,029,600	4,215,619	3,952,640
G + C content (mol%)	46.7	46	46.5	43.5	46.5
Total genes	3,884	3,877	4,002	4,426	3,907
rRNA	27	29	27	30	27
tRNA	86	88	86	86	84
Genomic Island	5	3	17	24	7
Prophage	2	2	6	4	5

**Fig 5:**
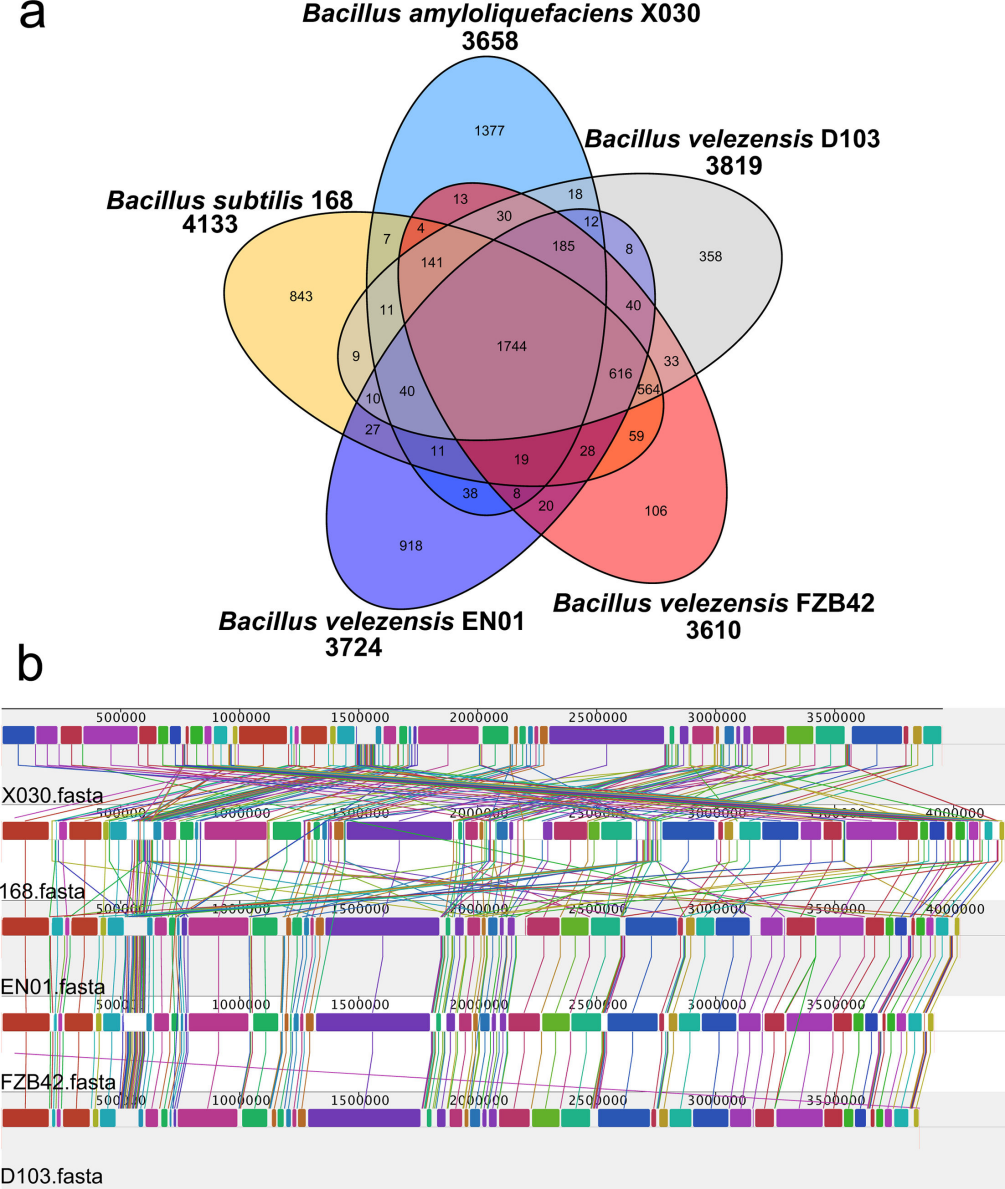
Genomic comparison of *B. velezensis* D103 with *B. velezensis* FZB42, *B. velezensis* EN01, *B. subtilis* 168, and *B. amyloliquefaciens* X030. (a) The Venn diagram illustrates the number of shared and unique clusters of orthologous genes among the strains. (b) Whole-genome comparison using Mauve progressive analysis, highlighting homologous regions, represented by color-coded boxes connected by lines. Forward and reverse regions are differentiated above and below the central axis.

Core-genome plot analyses of the five *Bacillus* genomes revealed a total of 1,744 genes across these species (Fig. 5a). The distribution showed that 3,135 genes were shared between D103 and *Bacillus subtilis* 168; 2,181 genes were shared between D103 and *Bacillus amyloliquefaciens* X030; and 2,655 genes were shared between D103 and *Bacillus velezensis* EN01. Additionally, 3,353 genes were common between D103 and *Bacillus velezensis* FZB42. D103 shared a considerable number of genes with *B. velezensis* FZB42 and *B. subtilis* 168, suggesting potential similarities with these strains as PGPR. Compared to the other four *Bacillus* strains, D103 contained 358 distinct genes. Among these, 335 genes were associated with assumed proteins and proteins with unknown function, while 21 genes were linked to proteins of known function, including terpene synthase (WP_077722691.1), damage-inducible protein DinB (WP_082998055.1), SAM-dependent methyltransferase (EYB36085.1), NUDIX hydrolase domain-containing protein (AJK64336.1), protein kinase, sporulation protein, and transcriptional regulator, as listed in Table S1.

To assess genetic relationships, whole-genome sequences of the five strains were analyzed using the Mauve program (Fig. 5b). The analysis revealed significant local collinear block (LCB) inversions and gene insertions or deletions in strain D103 relative to *Bacillus amyloliquefaciens* compared to X030 and *Bacillus subtilis* 168. However, D103 showed greater genetic similarity to *Bacillus velezensis* FZB42, with no significant insertions or deletions or LCB inversions observed compared to FZB42 and *Bacillus velezensis* EN01.

The annotation analysis of predicted amino acid sequences from strain D103 and four other *Bacillus* strains (FZB42, EN01, 168, and X030) using the dbCAN carbohydrate-active enzyme (CAZyme) database revealed that the D103 genome contains 193 CAZymes. These include 40 glycoside hydrolase (GH) enzymes, 34 glycosyltransferase (GT) enzymes, 17 carbohydrate esterases (CEs), three polysaccharide lyase (PL) enzymes, six auxiliary activities (AAs), and six carbohydrate-binding module (CBM) proteins (Table 4). Enzymes identified in D103, FZB42, EN01, and X030 involve acetylxylan esterase (CE family 6) (EC 3.1.1.72) and monooxygenases (AA family 10), such as lytic xylan monooxygenase/xylan oxidase (glycosidic bond-cleaving) (EC 1.14.99.-), lytic chitin monooxygenase (EC 1.14.99.53), lytic cellulose monooxygenase, lytic cellulose monooxygenase (C1-hydroxylating) (EC 1.14.99.54), and lytic cellulose monooxygenase (C4-dehydrogenating) (EC 1.14.99.56). These enzymes were found in D103, FZB42, EN01, and X030 but were absent in 168.While the numbers of CAZymes varied among strains, no CAZymes were discovered in D103, indicating similarities in their environmental habitat and nutritional sources. Differences in microbial ability to depolymerize and metabolize sugars may suggest ecological role differentiation to mitigate competition for resources (22).

**TABLE 4:** Comparative analysis of predicted carbohydrate-active enzyme families in *B. velezensis* D103, *B. velezensis* FZB42, *B. velezensis* EN01, *B. subtilis* 168, and *B. amyloliquefaciens* X030

CAZymes	*B. velezensis* D103	*B. velezensis* FZB42	*B. velezensis* EN01	*B. subtilis* 168	*B. amyloliquefaciens* X030
GH	40	41	42	58	42
GT	34	34	35	39	34
CE	17	17	17	18	17
PL	3	3	3	7	3
AA	6	6	6	5	6
CBM	6	6	6	14	6

**Fig 6:**
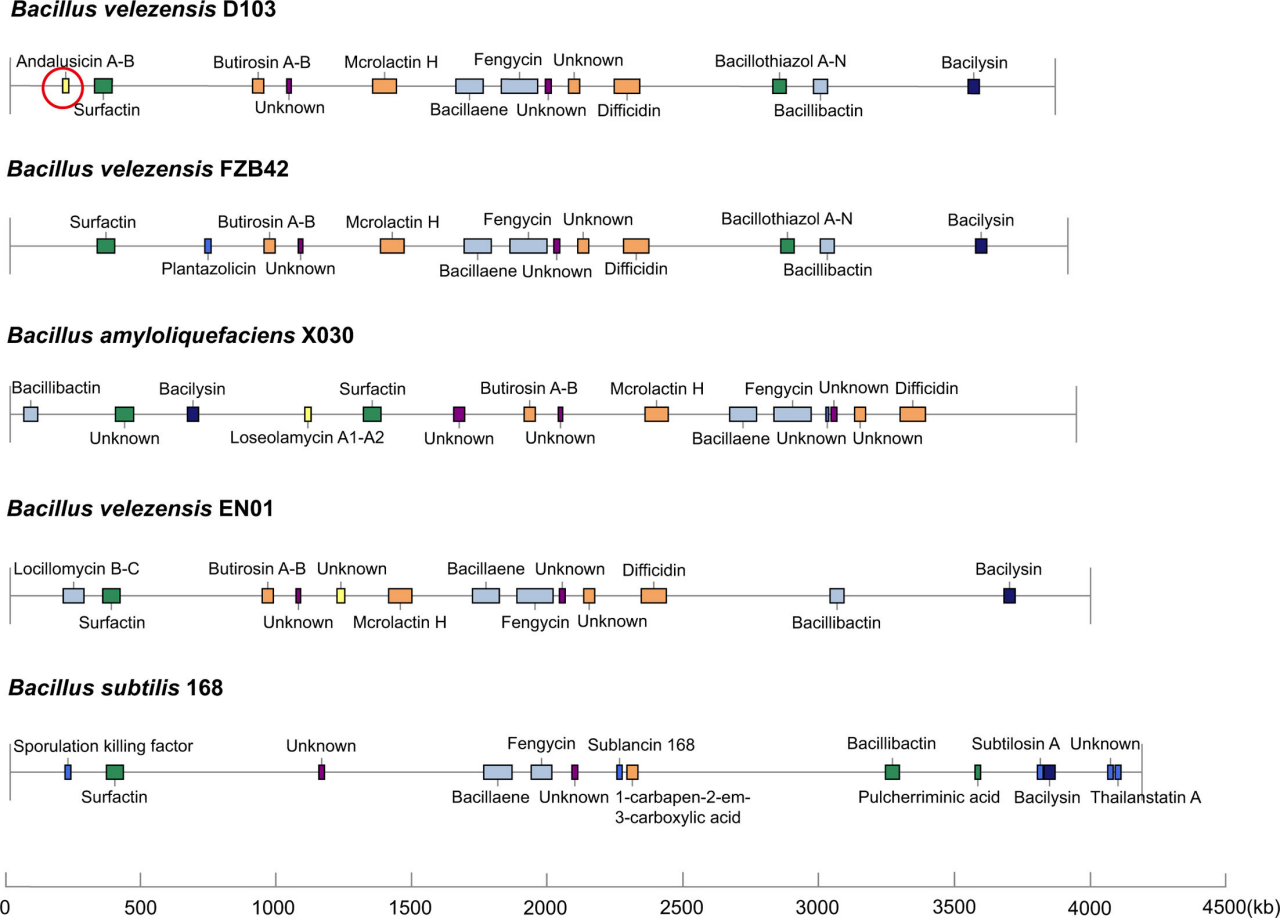
A comparative analysis of the locations of secondary metabolite gene clusters in *B. velezensis* D103, *B. velezensis* FZB42, *B. amyloliquefaciens* X030, *B. velezensis* EN01, and *B. subtilis* 168.

Genomic analysis of D103 using antiSMASH identified 13 secondary metabolite gene clusters: four NRPS, three TransAT-PKS, two terpenes, one lanthipeptide, and one other KS. These clusters are involved in the biosynthesis of various compounds, including andalusicin A/andalusicin B, surfactin, butirosin A/butirosin B, macrolactin H, bacillaene, fengycin, difficidin, bacillothiazol A-N, bacillibactin, and bacilysin (Table 5). Comparison of the secondary metabolite gene clusters in D103 with those in FZB42, X030, EN01, and 168 (Fig. 6) revealed that five clusters (2, 4–7) were present in each of the *Bacillus* strains. Additionally, four clusters (3, 5, 9, and 10) were shared by D103, FZB42, EN01, and X030; two clusters (12 and 13) were common to D103, FZB42, EN01, and 168; and one cluster (11) was found in both D103 and FZB42. Furthermore, three clusters (4, 8, and 9) were associated with unknown compounds. A distinctive cluster (1) responsible for the production of andalusicin A/andalusicin B was present only in D103.

**TABLE 5:** Comparative analysis of secondary metabolite gene clusters in *Bacillus velezensis* D103 and four other *Bacillus* strains (FZB42, X030, EN01, and 168)

D103		Gene cluster location				Presence (+) or absence (−)			
Cluster number	Type	From	To	Compound	Size (kb)	FZB42	EN01	168	X030
1	Lanthipeptide	193,743	216,358	Andalusicin A/andalusicin B	22,615	−	−	−	−
2	NRPS	311,522	376,328	Surfactin	64,806	+	+	+	+
3	PKS-like	894,710	935,954	Butirosin A/butirosin B	41,244	+	+	−	+
4	Terpene	1,020,841	1,037,485	Unknown	16,644	+	+	+	+
5	TransAT-PKS	1,337,888	1,425,686	Macrolactin H	87,798	+	+	−	+
6	TransAT-PKS, T3PKS, NRPS	1,645,193	1,745,891	Bacillaene	100,698	+	+	+	+
7	NRPS, TransAT-PKS, Betalactone	1,812,907	1,947,038	Fengycin	134,131	+	+	+	+
8	Terpene	1,975,295	1,997,178	Unknown	21,883	+	+	+	+
9	T3PKS	2,060,900	2,102,000	Unknown	41,100	+	+	−	+
10	TransAT-PKS	2,229,868	2,323,605	Difficidin	93,737	+	+	−	+
11	NRPS	2,815,113	2,864,622	Bacillothiazol A-N	49,509	+	−	−	−
12	NRPS, RiPP-like	2,965,190	3,016,986	Bacillibactin	51,796	+	+	+	−
13	Other	3,535,967	3,577,385	Bacilysin	41,418	+	+	+	−

### Plant growth-promoting genes in D103 strain

The D103 genome contains numerous genes predicted to be involved in plant growth-promoting activities (Table S2). Among these, moa clusters (*moaA-E*), responsible for encoding molybdenum cofactors, were identified, suggesting a possible role in nitrogen-fixing gene clusters or cofactors, essential for nitrogen assimilation (23). Additionally, D103 encodes critical elements such as sensor histidine kinase (*glnK*), a gene cluster for nitrate transport and reduction (*nasD-F*), HTH-type transcriptional regulators (*tnrA* and *glnR*), glutamine synthetase (*nifS*), ammonium transporter (*nrgA*), nitrogen regulatory PII-like protein (*nrgB*), a gene cluster for urease subunit (*ureA-C*), a gene cluster for nitrate reductase (*narG-J*), a probable transcription regulator (*arfM*), and nitrite extrusion protein (*narK*). These components contribute to facilitating nitrogen assimilation (24). The genome of D103 revealed the presence of 19 phosphatase genes involved in phosphorus solubilization. Additionally, D103 contained potassium transporter genes, including K^+^/H^+^ antiporter subunits (*khtS-U*), ktr system potassium uptake proteins (*ktrA*, *ktrC*, and *ktrD*), and putative potassium channel protein (*yugO*) (25). Furthermore, the D103 genome included magnesium transporter genes (*mgtE*, *corA*) (26), manganese assimilation-related genes (*mntH*, *mntR*, and *mntP*) (27), iron assimilation-related genes (*yclQ*, *yusV*, and *yvrA-C*) (28), and a cluster of bivalent cation assimilation-related genes (*tuaA-H*). These genes were hypothesized to play crucial roles in mineral element uptake and the detoxification of heavy metal ions in both bacteria and plants.

PGPR produce VOCs that have the potential to serve as environmentally friendly alternatives to chemical fertilizers. These compounds, including 2,3-butanediol, enhance plant growth by improving nutrient availability, inducing metabolic activities, and stimulating defense responses (29). Analysis of the D103 genome revealed the *als* gene cluster (*alsD*, *alsS*, and *alsR*) and (R,R)-butanediol dehydrogenase (*bdhA*), key components of the biosynthetic pathway for 2,3-butanediol from pyruvate (30). Additionally, we identified the *acu* gene cluster (*acuA-C*) responsible for encoding acetoin, a compound known to promote the development of ISR in plants (31).

The genome of D103 contains genes involved in trehalose biosynthesis (*treP*, *treA*, and *treR*), spermidine and polyamine biosynthesis (*speA*, *speH*, *speB*, *speE*, and *msmX*), and siderophore biosynthesis (*dbhA*, *dbhB*, *dbhC*, *dbhE*, and *dbhF*). These gene clusters contribute to promoting plant growth and inhibiting the growth of plant pathogens (32–34). Additionally, D103 contains 12 genes involved in IAA biosynthesis, utilizing pathways such as indole-3-acetonitrile (*yhcX*, *trpA-F*, *trpP*, and *trpS*) and indole-3-pyruvate (*dhaS*) for IAA synthesis (35). Furthermore, the genome includes genes related to auxin excretion (*ywkB*) and IAA acetylation (*ysnE*), indicating involvement in the tryptophan-independent IAA biosynthetic pathway (19). The presence of phytase genes (*phy*) suggests the potential to degrade phytate, thereby promoting plant growth under phosphate-limited conditions (31).

Effective biofilm production by PGPR enhances their adherence to plant roots and augments plant growth-promoting activities (36). Flagella, motility, and chemotaxis play important roles in all stages of biofilm formation (37). D103 possesses genes associated with bacterial chemotaxis (*che* gene cluster and *mcpA-C*), flagellar assembly (*fli* cluster, *flg* cluster, *flh* cluster, *motAB*), and swarming motility (*swrB-D* and *swrAA*, *efp*). Genes involved in the initial stages of biofilm formation, including histidine kinases (*kinA-D*), master regulators (spo0A, spo0B, spo0E, spo0F, and spo0J), and transcriptional regulators (17 genes), were identified in the genome of D103. Components of the biofilm matrix, including secreted proteins (TasA, TapA, and BslA), mineral scaffolds, extracellular DNA, and extracellular polysaccharides (*eps* gene cluster: *eps*C-O), were also present. Additionally, genes related to biofilm surface formation (*yua*B), colony biofilm strength (*pgs*A), and the development of multicellular communities (*ecs*B, *ylb*F, *ymc*A, and *yqe*K) were identified in the D103 genome (38).

Based on comparative genomic analyses (Table S3), 199 PGP genes were identified in the D103 genome. *Bacillus velezensis* FZB42 and *Bacillus subtilis* 168 shared the most PGP genes with D103, 198 and 195, respectively. Notably, FZB42 was devoid of *sigW*, a gene involved in the transcriptional regulation of biofilm formation, while 168 was devoid of *ybjI*, a gene associated with phosphorus assimilation; *tuaA*, a gene for divalent cation assimilation; *trpC*, a gene for IAA biosynthesis; and swarming motility gene *swrAA. Bacillus velezensis* EN01 contains 153 PGP genes shared with D103, whereas *Bacillus amyloliquefaciens* X030 exhibits the lowest number of PGP genes with D103, with only 126 shared genes. Compared to other strains, fewer genes associated with swarming motility, flagellar assembly, and biofilm formation were identified in the genomic comparisons between D103 and both EN01 and X030.

### Plant growth-promoting capacity of strain D103

**Fig 7:**
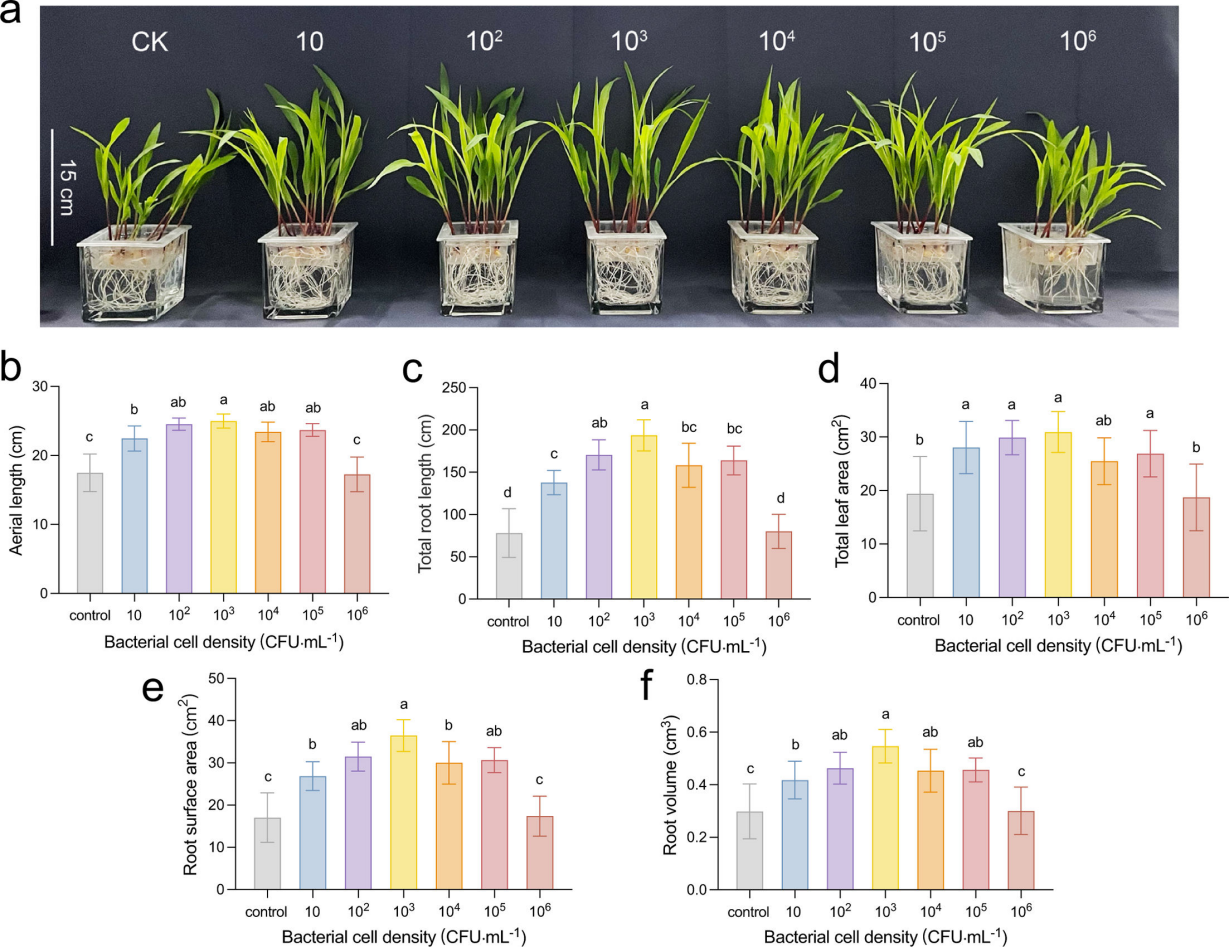
Hydroponic cultivation was employed to assess the growth promotion of maize by applying varying concentrations of the *B. velezensis* D103 strain. (a) Maize growth responses were observed across a range of D103 strain concentrations, from 10 to 10^6^ CFU·mL^−1^, with the control (CK) lacking the D103 strain culture. The agronomic characteristics of maize seedlings were quantified, including (b) aerial length, (c) total root length, (d) total leaf area, (e) root surface area, and (f) root volume. Different letters indicate statistically significant differences between treatments (*P* < 0.05, *n* = 10).

In a hydroponic cultivation system, maize plants were exposed to a range of D103 cell suspension concentration from 10 to 10^6^ CFU·mL^−1^. Growth parameters assessed included shoot length (aerial parts), total leaf area, and both fresh and dry weights of the aerial parts. Root development was evaluated based on total root length, surface area, volume, and fresh and dry weight. The results demonstrated a significant impact of D103 cell suspension concentrations on maize plant growth (Fig. 7a; Fig. S4). Specifically, maize seedlings treated with a 10^3^ CFU·mL^−1^ D103 cell suspension exhibited significant increases in shoot length and total leaf area, showing 43% and 60% enhancements (*P* < 0.001), respectively, compared to the control group. Additionally, these seedlings demonstrated superiority with total root length reaching 193.62 cm (148% higher than the control), a root surface area of 36.46 cm^2^ (114% higher than the control), and a root volume of 0.54 cm^3^ (86% higher than the control) (Fig. 7b through e). Moreover, the dry weights of both aerial parts and roots of maize treated with the 10^3^ CFU·mL^−1^ D103 cell suspension were significantly increased compared to the control (*P* < 0.001) (Fig. S5). These findings indicate that a D103 cell suspension concentration of 10^3^ CFU·mL^−1^ is optimal for enhancing maize plant growth and development.

## DISCUSSION

The application of PGPR as biofertilizer represents a viable approach for advancing sustainable agriculture intensification (39). The isolation of *Bacillus* strain D103 from maize rhizosphere soil, combined with phenotypic and phylogenetic analyses, established its classification as *Bacillus velezensis*. This specie is distinguished by its resilience to adverse environmental conditions, secretion of diverse hydrolytic enzymes, enhanced plant growth, exertion of antagonistic effects on phytopathogens, and maintenance of a favorable safety profile, highlighting its significant agricultural potential (40, 41). Accordingly, *Bacillus velezensis* D103 demonstrates potential as a PGPR for agricultural applications. This investigation involved a comprehensive characterization of D103 to evaluate its efficacy in promoting plant growth.

Nitrogen is an essential element for plant growth, and nitrogen-fixing bacteria play a crucial role in fixing atmospheric nitrogen into a form that can be utilized by plants, thereby enhancing the soil nitrogen reservoir (5). In this study, *Bacillus velezensis* D103 demonstrated nitrogen-fixing capabilities and thrived in nitrogen-deficient environments. Previous research has established that nitrite reductase, a crucial enzyme nitrogen fixation, is encoded by the *nas* operon. This operon activates transcription by binding to motifs within the TnrA promoter, thereby enhancing the conversion of nitrite, nitrate, and urea into ammonium (42). Twenty-four nitrogen assimilation genes including *nasDEF* and *tnrA* were identified in the D103 genome. Additionally, D103 demonstrated ammonia production, a primary nitrogen source for plants, consequently promoting plant growth. The nitrogen-fixing potential of D103 was demonstrated by acetylene reduction analysis assay.

Phosphorus is a crucial macronutrient for plant growth and development. Due to its rapid immobilization, rendering it inaccessible to plants, phosphate-solubilizing bacteria are essential for converting insoluble phosphorus into bioavailable forms (43). Strain D103 demonstrated the ability to solubilize inorganic phosphorus, thereby enhancing phosphorus availability for plants. Nineteen genes associated with phosphate solubilization, including *pstBS* and *phoADEPR*, were identified in strain D103. The *pst*, a phosphate transport system permease, potentially increases phosphate uptake and bioavailability under phosphate-limited conditions (44). Similarly, Torres et al. (45) reported the presence of the organic phosphorus mineralization gene (*phoACDX*) and the P-starvation response regulatory gene (*phoBPR*) in the *Bacillus sensu lato* genome. The phosphorus assimilation gene in D103 increases phosphate utilization in the host plant.

Auxin, particularly IAA, is a critical regulator of plant growth and development, influencing numerous processes, including seedling growth and root formation (46). Previous research has shown that inoculation with IAA-producing *Bacillus amyloliquefaciens* can promote lateral root growth, elongation, and root hair formation in plants such as *Arabidopsis thaliana* (47). This study demonstrated that D103 exhibited IAA production, potentially stimulating root development in the host plant. Notably, plant roots release tryptophan in the rhizosphere, serving as a substrate for IAA biosynthesis by rhizobacteria (48). In this study, 12 genes identified in the D103 genome were putatively involved in IAA biosynthesis, including *dhaS*, encoding aldehyde dehydrogenase, an enzyme crucial for converting indole-3-acetaldehyde to IAA in the indole-3-pyruvic acid (IPyA) pathway. Studies on *Bacillus amyloliquefaciens* SQR9 have demonstrated that mutation of the *dhaS* gene reduces IAA production to only 23% of wild-type levels (49). These findings suggest that the IPyA pathway is a significant contributor to IAA biosynthesis in D103. Furthermore, D103 possessed genes for synthesizing other potentially beneficial hormones, including trehalose, and phytase. Similar to findings in other bacterial strains, these hormones are implicated in promoting plant growth and enhancing plant tolerance to diverse environmental stresses (50, 51).

Iron, which typically presents in soil as insoluble trivalent Fe^3+^ hydroxide, is not readily assimilated by plants. Siderophores, produced and secreted by bacteria, facilitate the uptake of iron into plant cells (52). The *dhbA-F* operon plays a crucial role in siderophore biosynthesis. Studies have shown that mutants lacking the *ΔdhbA* gene are unable to dehydrogenate (2S,3S)-2,3-dihydroxy-2,3-dihydrobenzoate to 2,3-dihydroxybenzoate (DHB) (53). This inability disrupts extracellular electron transport with ferric iron captured by DHB. This study revealed that D103 possesses the ability to produce siderophores and contains a *dhb* cluster in its genome. Notably, the siderophores produced by D103 contributed to biocontrol by competing for iron, thereby reducing its availability to pathogens (53). The results demonstrated that D103 enhanced Fe^3+^ availability under iron-deficient conditions, which was a key factor promoting maize seedling growth.

Colonization of the plant rhizosphere by bacterial strains is the initial and most crucial step in promoting plant growth and health. Genes associated with motility, chemotaxis, adhesion, and biofilm formation are believed to contribute to colonization (54). While swimming has been identified as the primary mechanism for movement in liquid media, swarming is considered more significant in natural soil environments. In these conditions, dynamic multicellular rafts are formed as groups of cells that move rapidly across solid surfaces, potentially enhancing nutrient acquisition. This phenomenon has been observed in *Bacillus amyloliquefaciens* T-5 colonization nutrient-rich tomato roots (55, 56). The study highlighted the swimming, swarming, and biofilm formation abilities of strain D103, and identified a large number of genes in its genome associated with bacterial chemotaxis, flagellar assembly, swarming motility, and biofilm formation. Bacteria forming biofilms have been reported to exert more beneficial effects on plant growth compared to their planktonic cells counterparts. For instance, *Pseudomonas azotoformans* FAP5 contributes to root colonization and improves wheat performance under stressful conditions (57, 58). Therefore, these characteristics are essential for D103 adaptation, persistence under varying environmental conditions, and the promotion of maize growth.

Biological control of rhizosphere microbes provides host plants natural protection against pathogens (59). The production of secondary metabolites by antagonistic bacteria is the primary mechanism of disease suppression (60). In this study, we identified 13 secondary metabolite gene clusters with antimicrobial activity in the genome of D103. Combined with observed antifungal results, these findings suggested the significant biocontrol potential of D103 as an inoculum. This outcome aligns with previous reports emphasizing the secondary metabolite potential of *B. velezensis* strain (61). Notably, D103 contained a unique cluster of genes responsible for synthesizing andalusicin A and andalusicin B, a novel family of class III lantibiotics derived from *Bacillus thuringiensis* subsp. *andalousiensis* NRRL B23139. This cluster demonstrated biological activity directed at *Bacillus cereus* and related species (62). These findings emphasize the promising potential of D103 for managing plant pathogens in agricultural applications.

Finally, the direct impact of D103 on plant growth was evaluated, revealing a significant enhancement in maize growth when applied at the appropriate concentration. Numerous studies have demonstrated that PGPR stimulate plant root growth and increase root fresh and dry weight, thereby enhancing nutrient uptake (63, 64). D103 increased the length and surface area of corn root structures, offering benefits for improved ion uptake and nutrient storage. Similarly, other strains of *Bacillus velezensis*, such as SQR9, have been shown to promote cucumber growth through various PGP mechanisms (65). Collectively, these results suggest that D103 has potential as both a maize plant growth promoter and a natural biocontrol agent against plant pathogens.

### Conclusion

**Fig 8:**
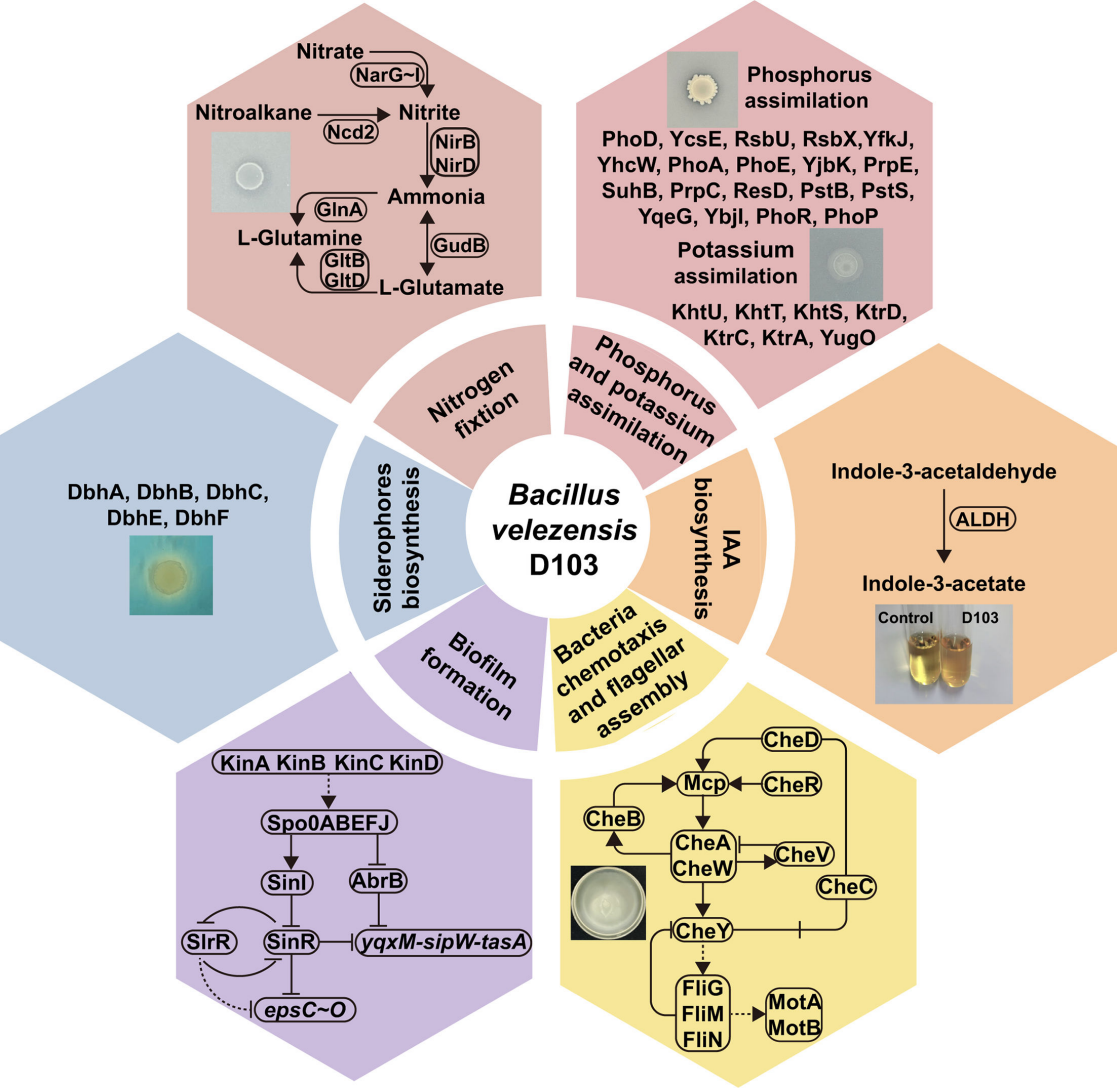
*Bacillus velezensis* D103 possesses beneficial metabolic pathways and genes associated with plant growth-promoting rhizobacteria.

*Bacillus velezensis* D103 was evaluated for its plant growth-promoting properties, demonstrating its potential to increase maize plant height and enhance root development. Genomic analyses further validated the findings from *in vitro* assays and hydroponic plant experiments, confirming the efficacy of D103’s role as PGPR. The genomic analysis revealed the presence of a multitude of genes potentially involved in plant growth promotion. Collectively, these findings strongly suggest that D103 is a promising candidate for biofertilizer development, with potential application as a single inoculant or as part of a microbial consortium (Fig. 8)

## MATERIALS AND METHODS

### Isolation and culture conditions of bacteria

Soil samples were collected from the rhizosphere of maize in an experimental field at the Haicheng Branch Campus of Shenyang Agricultural University in Haicheng, China (latitude 40°98′08 N, longitude 122°72′64 E). The field was situated at an elevation of 14.3 m above sea level and had been cultivated with maize crop for >5 years. The area experiences extreme annual temperature variations (−30°C to 34.4°C) and an average annual rainfall of 652 mm. Soil samples were collected in June 2020, and the maize rhizosphere bacterium D103 was isolated using the gradient dilution technique to assess its potential for promoting plant growth. The bacterial strain was cultured in Luria-Bertani (LB) medium, composed of 0.5% yeast extract, 1% sodium chloride, and 1% peptone, with 1.5% agar added for solidification. For long-term preservation, pure cultures were stored at −80°C in an LB medium supplemented with 20% glycerol (vol/vol).

### Identification of the strain

Gram staining and spore morphology of the bacterial strain were examined microscopically. The 16S rRNA gene was subjected to colony polymerase chain reaction (PCR) using forward primer 27F (5′-AGAGTTTGATCCTGGCTCAG-3′) and reverse primer 1492R (5′-GGTTACCTTGTTACGACTT-3′) (66). The PCR cycling parameters were as follows: initial denaturation at 94°C for 3 min, followed by 30 cycles of denaturation at 95°C for 40 seconds, annealing at 56°C for 40 seconds, and elongation at 72°C for 90 seconds, with a final extension step at 72°C for 10 min. Purified PCR products were sequenced using an ABI 3730 Genetic Analyzer (Applied Biosystems). The obtained sequences were compared with reference sequences from the NCBI database using Basic Local Alignment Search Tool for Nucleotides software. Reference 16S rRNA sequences were retrieved from GenBank. Sequence alignment was performed using MAFFT (https://mafft.cbrc.jp/alignment/server/). A phylogenetic tree was generated using FastTree (v.2.1.7) (67) with 1,000 bootstrap replicates, and the tree was visualized using ITOL online tool (https://itol.embl.de).

### Assessment of *in vitro* plant growth-promoting capacity

To evaluate the nitrogen-fixing capability of strain D103, Ashby’s nitrogen-free agar medium was utilized (68). The ability of D103 to grow on this medium indicated its nitrogen-fixing potential. Nitrogenase activity was quantified using the acetylene reduction assay and gas chromatography (GC) as described by Swamy et al. (69). Briefly, strain D103 was inoculated into nitrogen-free Ashby’s medium and incubated for 48 h at 37°C with shaking at 180 rpm. Subsequently, 2 mL of the culture was transferred to 45 mL of Ashby semisolid medium for further incubation. Afterwards, 25 mL of the enriched culture was transferred to 100-mL vials. A sterile syringe replaced 10 mL of air with equivalent acetylene gas and was incubated for 24 h. Nitrogen fixation was assessed by measuring ethylene production using the acetylene reduction assay. The ethylene produced was analyzed using gas chromatography (Shimadzu GC 2010PLUS, Japan) equipped with a flame ionization detector. The chromatographic column used was packed with alumina (3-m length, 0.53-mm diameter). The GC conditions were as follows: oven temperature was maintained at 80°C, while the injector and detector temperatures were set at 165°C. Nitrogen gas and hydrogen gas flow rates were each 40 mL·min^−1^, and the air flow rate was 45 mL·min^−1^. A 1-mL gas sample was injected, and the peak area for ethylene was measured in relation to a standard ethylene. Ethylene production was expressed as nanoliters of ethylene formed per milliliter of medium per hour at 37°C.

To assess the phosphorus-solubilizing potential of strain D103, modified Pikovaskaia’s agar medium was utilized (70). The inoculated plates were incubated at 37°C for 7 days. Phosphorus solubilization was assessed by observing the formation of clear zones around the colonies. The amount of solubilized phosphorus was quantified using the phosphomolybdate method (71).

To assess the potassium-solubilizing capability of strain D103, the Aleksandrov agar medium was used (72). The plates inoculated with the strain, were incubated at 37°C for 7 days. The potassium-solubilizing potential of D103 was determined by observing the formation of clear zones around the colonies, which indicated positive potassium solubilization.

IAA production was assessed using the method described by Mahdi et al. (73), involving the application of Salkowski’s reagent. A color change from yellow to pink indicated IAA production. Quantification of IAA was carried out by UPLC following the method described by Shah et al. (74). Briefly, D103 was inoculated into 500 mL of LB medium supplemented with 0.5% L-tryptophan and incubated at 37°C with shaking at 180 rpm for 7 days. The culture liquid phase was centrifuged at 6,500 rpm for 10 min, acidified to pH 2.5–3.0 with 1-N HCl, and extracted with an equal volume of ethyl acetate. The ethyl acetate phase was evaporated under vacuum using a rotary evaporator at 40°C, and the residue was redissolved in 1 mL of methanol and stored at −20°C. UPLC analysis was performed using a Waters H-Class system (Milford, MA, USA) with a C18 column (5 µm, 4.6 × 250 mm). The mobile phase consisted of a water-methanol mixture (60:40) with 0.5% acetic acid, and elution was conducted at a flow rate of 1 mL·min^−1^. IAA was detected with a UV-visible detector at 280 nm. Retention times and peak areas were determined using standard IAA calibration curves.

### Ammonia and siderophore production, surface motility, and biofilm formation

Bacterial cultures were assessed for ammonia production using Nessler’s reagent, as per outlined by Oliva et al. (75). A mixture of 5 mL of supernatant from centrifugation of overnight culture broth with 1 mL of Nessler’s reagent indicated NH_3_ production by a color change from yellow to brown. Siderophore production was determined using CAS medium (76). Inoculated plates were incubated at 37°C for 4 days, and the presence of an orange-yellow halo around colonies confirmed positive siderophore production.

To assess surface motility, 2.5 µL of each strain cultured overnight (1 × 10^8^ cells) was spotted at the center of LB plates solidified with 0.3% and 0.7% agar. Swimming motility and swarming motility over the surface were analyzed on separate plates (90 mm) (77). Incubation occurred at 37°C for 24 h, with colony diameters measured at 6-h intervals.

Biofilm formation was evaluated in 96-well polystyrene microtiter plates using the crystal violet method (78). OD_590 nm_ values served as an index of biofilm formation. EPS from strain D103 was extracted using the EDTA extraction method (79). Polysaccharides within the EPS extracts were quantified using a modified phenol-sulfuric acid colorimetric method, employing glucose as the standard (80). Protein content in the EPS extract was determined using the Coomassie brilliant blue staining method, with bovine serum albumin as the standard (81). Nucleic acids were quantified using the diphenylamine method (82).

### Detection of *in vitro* hydrolytic activity

To assess *in vitro* hydrolytic activity, we investigated the production of cellulose (83), protease (84), amylase (85), and β-1,3-glucanase (86) by strain D103, as previously outlined. We quantified the activities of these four enzymes using the Microorganism Cellulase ELISA Kit, Microorganism Protease ELISA Kit, Microorganism Amylase ELISA Kit, and Microorganism β-1,3-glucanase ELISA Kit, all provided by Jiangsu Meimian Industrial Co., Ltd. Additionally, ACC deaminase production and enzyme activity were directly determined using the ACC deaminase ELISA Kit from Jiangsu Meimian Industrial Co., Ltd.

### *In vitro* antagonism assay

To assess the antagonistic effectiveness of D103 against phytopathogens, we selected five fungal pathogens: *Fusarium graminearum*, *Athelia rolfsii*, *Fusarium thapsinum*, *Gibberella fujikuroi*, and *Gibberella moniliformis*. The antifungal resistance of D103 was determined through a triple-replicated dual culture experiment (23). In this experimental setup, strain D103 and each pathogen were simultaneously cultured on 9-cm potato dextrose agar plates, with 8-mm plugs of the pathogen positioned 3.5 cm apart. The plates were then incubated at 28°C for duration of 4 days.

### DNA extraction

Genomic DNA extraction was performed using the cetyltrimethylammonium bromide method (87).The DNA was subsequently assessed for concentration, quality, and integrity using a Qubit Fluorometer (Invitrogen, USA) and a Nano Drop Spectrophotometer (Thermo Scientific, USA).

### Genome sequencing and assembly

Qualified genomic DNA was fragmented with G-tubes (Covaris, Woburn, MA, USA) and subjected end repair to prepare SMRTbell DNA template libraries with fragment size of>10kb using the blue pippin system, following the manufacturer’s specification (PacBio, Menlo Park, CA, USA). The quality of the libraries was assessed using Qubit (v.2.0) Fluorometer (Life Technologies, CA, USA), and the average fragment size was determined with a Bioanalyzer 2100 (Agilent, Santa Clara, CA, USA). SMRT sequencing was performed on the Pacific Biosciences Sequel (PacBio) according to standard protocols.

Qualified genomic DNA was first randomly sonicated, followed by end repair, A-tailing, and adaptor ligation using the NEBNext ΜLtra DNA Library Prep Kit for Illumina (NEB, USA) in accordance with the manufacturer’s protocol. DNA fragments ranging from 300 to 400 bp were selectively enriched by PCR. The PCR products were subsequently purified with the AMPure XP system (Beckman Coulter, Brea, CA, USA). Library size distribution was assessed using a 2100 Bioanalyzer (Agilent), and concentration was determined by real-time PCR. Genome sequencing was performed on the Illumina Novaseq 6000 sequencer utilizing paired-end technology (PE 150).

Continuous long reads obtained from SMRT sequencing were utilized for *de novo* assembly using Falcon (v.0.3.0) (88). Raw data from Illumina platform were processed with FASTP (v.0.20.0) (89) by applying the following standards: (i) reads containing ≥10% unidentified nucleotides (N) were discarded; (ii) reads with ≥50% bases having phred quality scores of ≤20 were excluded; and (iii) reads aligned to the barcode adapter were removed. Following filtration, the resulting clean reads were employed to refine the genome assembly and enhance accuracy. The final genome sequences were determined using Pilon (v.1.23) (90) to correct errors and ensure high-quality assembly.

### Genome component prediction

Open reading frames were predicted using the NCBI prokaryotic genome annotation pipeline (91). rRNAs were identified using rRNAmmer (v.1.2) (92), while tRNAs were detected with tRNAscan (v.1.3.1) (93). Small RNAs (sRNAs) were identified with cmscan (v.1.1.2) (94).

### Genome composition prediction and annotation

The genome was functionally annotated using several databases, including the Non-redundant Protein Database, GO, KEGG, COG, and Swissprot. To provide a comprehensive overview of the genomic data, CGview (version 2.0.2) (95) was utilized.

### Analysis of average nucleotide identity

The ANI of the D103 genome was compared with 41 sequenced *Bacillus* strain genomes FastANI tool (v.1.1) (96). Clustering was performed using bactaxR (v.3.6.1) package in R (97). The phylogenetic tree was generated with ggtree (v.1.16.6) (98), and a heat map was produced using TBtools-II (v.21.0.3) (99). A total of 41 *Bacillus* reference whole-genome sequences were obtained from GenBank for this analysis.

### Comparative genomics

In the comparative genomics analysis, four *Bacillus* strains (*Bacillus velezensis* FZB42, *Bacillus velezensis* EN01, *Bacillus subtilis* 168, and *Bacillus amyloliquefaciens* X030) served as reference strains. Genomic characterization utilized NCBI annotated data. Gene families derived from protein sequences of both reference and target bacteria were analyzed using orthoMCL software (v.2.0.8) , resulting in Venn diagrams (100). Genomic comparisons of the five *Bacillus* strains employed Mauve software (v.2.3.1) (101). Genomic islands were identified using the Island Viewer four online platform, while prophages were predicted with the PHASTER online tool (http://phaster.ca/).

### Carbohydrate-active enzyme identification

To identify genes encoding CAZymes in the D103 genome, the dbCAN3 database (http://bcb.unl.edu/dbCAN2/) was utilized. This analysis detected various CAZyme categories, including GH, PL, CE, GT, AA, and CBM (102).

### Secondary metabolite analysis

To analyze secondary metabolites and identify biosynthetic gene clusters associated with the production of antimicrobial compounds from various chemical classes, the antiSMASH online tool (https://antismash.secondarymetabolites.org) was employed.

### Functional gene analysis related to plant growth promotion

The D103 genome was investigated for genes associated with PGP, including those involved in mineral assimilation, IAA synthesis, bacterial chemotaxis, flagellar assembly, and biofilm formation. The sequences identified were compared with the reference sequences in GenBank to validate their accuracy. The PGP-related genes from the D103 genome were compared at the amino acid level with those in the genomes of *Bacillus* strains: FZB42, EN01, 168, and X030 using the NCBI database.

### Growth-promoting capacity of D103 on maize

To assess the growth-promoting capabilities of D103 on maize, a bacterial suspension was prepared by incubating D103 for 12 h, resulting in an optical density of OD_590 nm_ = 1 (1 × 10^8^ CFU·mL^−1^). The suspension was serially diluted with sterile water to achieve final concentrations of 10^6^, 10^5^, 10^4^, 10^3^, 10^2^, and 10 CFU·mL^−1^. Maize seeds were sterilized in 75% ethanol for 2 min followed by 1% sodium hypochlorite for 10 min, and subsequently washed four to five times with sterile water. These seeds were soaked in the different concentrations of bacterial suspensions for 12 h, placed on filter paper in petri dishes with a small amount of distilled water, and allowed to germinate for 3–5 days in the dark at 24°C. Control seeds treated with sterile water were included, and each treatment consisted of three replicates of 10 plants each. Germinated seeds were transferred to the Hoagland hydroponic cultivation system, containing the respective concentrations of the bacterial suspension. The hydroponic solution was replaced every 2  days. All samples were incubated in an artificial climate room mimicking natural maize growing conditions (18°C at night, 24°C during the day, and 65% relative humidity).

After 2 weeks, several parameters were assessed and recorded to evaluate maize seedling growth. These parameters included aerial length, aerial dry weight, and root dry weight. Additional metrics such as root length, root volume, and root surface area were measured using the Root System Analyzer (WinRHIZO, Regent Instruments Inc., Canada). The total leaf area was determined with the Portable Area Meter Model LI-3000C (Li-Cor, Lincoln, NE, USA).

### Statistical analysis

Statistical comparisons among treatments were performed using one-way analysis of variance in GraphPad Prism 9 (v.9.5.1). The Shapiro-Wilk test was utilized to evaluate the normality of the data. Results are expressed as the mean ± standard deviation from three independent replicates. Statistical significance was defined as *P* < 0.05.

## Data Availability

The gene sequence of *Bacillus velezensis* D103 is available at the NCBI website with accession number CP095093. The URL of the gene sequence is https://www.ncbi.nlm.nih.gov/nuccore/CP095093.

## SUPPLEMENTAL MATERIAL

Supplemental figures spectrum.01147-24-s0001.docx Fig. S1 to S5.

Fig. S6 spectrum.01147-24-s0002.tif Mechanism of *Bacillus velezensis* D103 as a PGPR to promote the growth of maize seedlings.

Table S1 spectrum.01147-24-s0003.xlsx Unique genes with known functions in *B. velezensis* D103 compared to the other four *Bacillus* strains (FZB42, X030, EN01, and 168).

Table S2 spectrum.01147-24-s0004.xlsx Prediction of genes or gene clusters associated with plant growth-promoting activities in the genome of *Bacillus velezensis* D103.

Table S3 spectrum.01147-24-s0005.xlsx Comparative analysis of plant growth-promoting genes in D103, FZB42, EN01, 168, and X030.

ASM does not own the copyrights to Supplemental Material that may be linked to, or accessed through, an article. The authors have granted ASM a non-exclusive, world-wide license to publish the Supplemental Material files. Please contact the corresponding author directly for reuse.
