# Supplementary material for: Growth promotion on maize and whole-genome sequence analysis of Bacillus velezensis D103
Source: Microbiol Spectr. 2024 Nov 7;12(12):e01147-24. doi: 10.1128/spectrum.01147-24 (PMC11619478; doi:10.1128/spectrum.01147-24)
Supplement: Supplemental figures — Fig. S1 to S5. [file spectrum.01147-24-s0001.docx]

**Supplementary Figures**

**Fig. S1** ANI analyses of 42 *Bacillus*. Redder colors indicate higher similarity. Red color indicates high ANI value and light blue color indicates low ANI value. ****

**Fig. S2** The chromatogram of IAA was quantified by UPLC. (a) Concentration of 0.2 mg·L^-1^ IAA standard, elution retention time of 3.69 min; (b) chromatogram of strain D103 extract diluted 100 folds, elution retention time of 3.69 min.


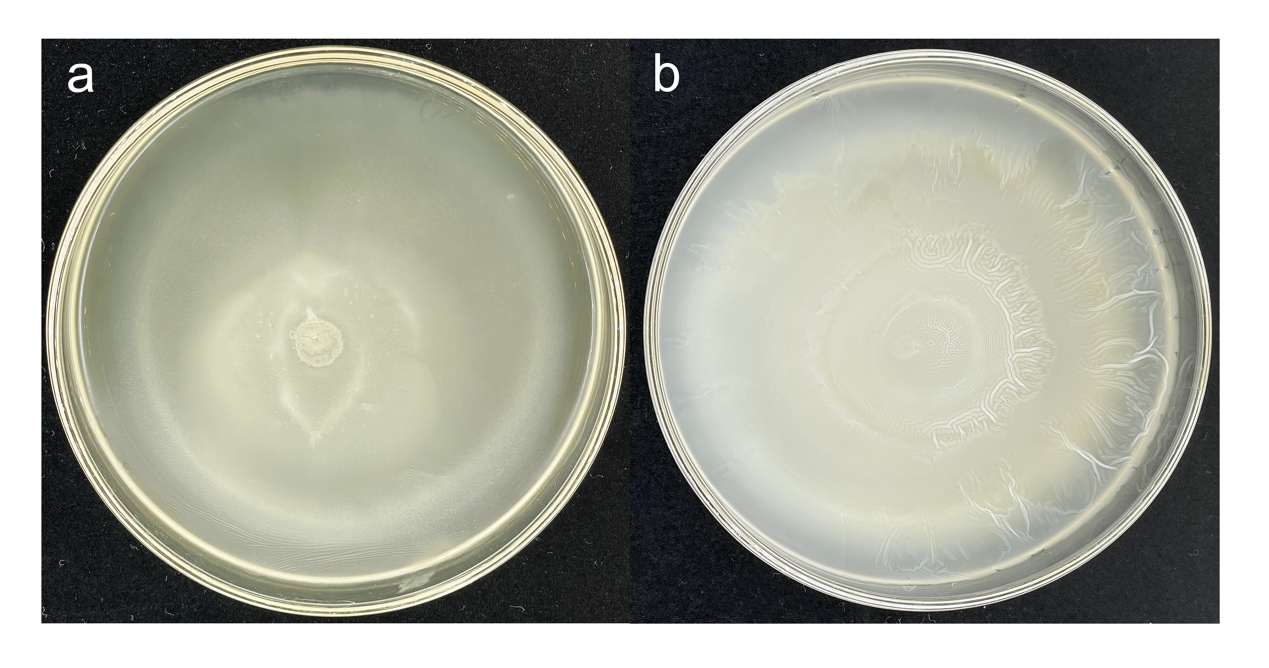


**Fig. S3** (a) The swim motility of D103 was assessed by incubating it on 0.3% agar plates at 37 ℃ for 12 hours. (b) The swarming motility of D103 was examined on 0.7% agar plates, and the swarm motility was observed after incubation at 37 ℃ for 16 hours.


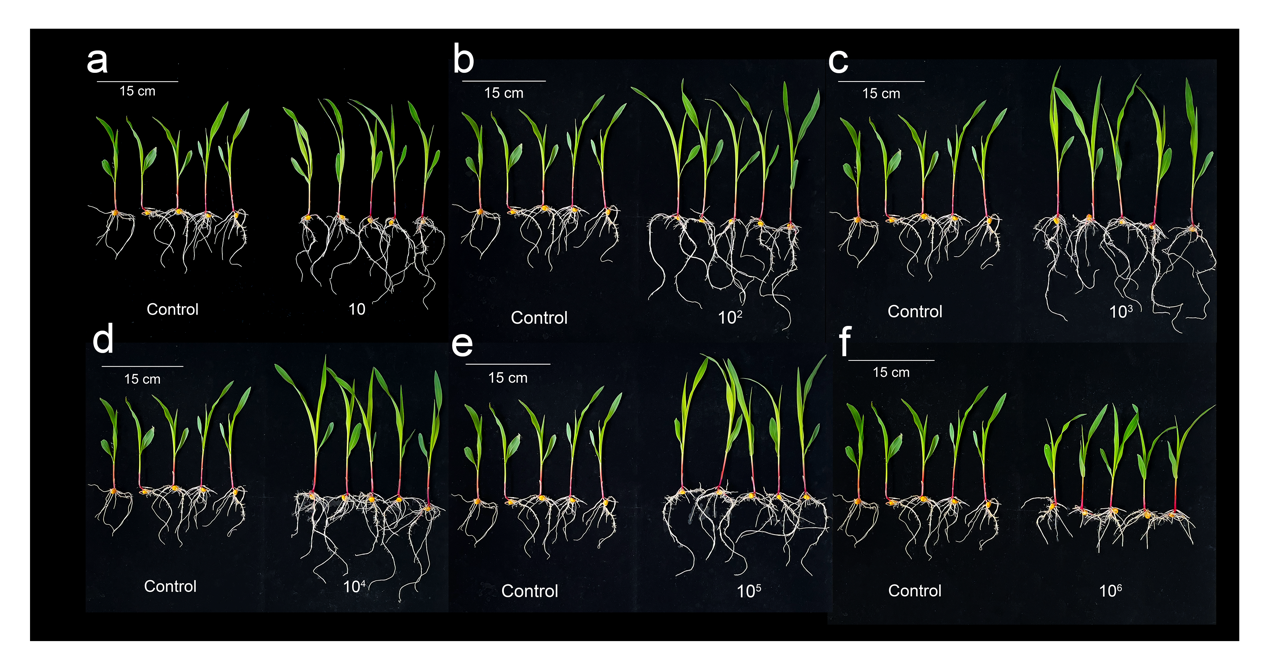


**Fig. S4** The growth of maize seedlings was assessed in cultures containing strain D103 at various concentrations, ranging from (a-f) 10 to 10^6^ CFU^.^mL^-1^.Top of Form

**Fig. S5** Agronomic attributes of maize seedlings were evaluated in cultures with varying concentrations of strain D103, comprising (a) aerial dry weight and (b) root dry weight. Different letters indicate statistically significant differences between treatments (*P* < 0.05, n = 10).Top of Form
