# Supplementary figures and images for: Growth promotion on maize and whole-genome sequence analysis of Bacillus velezensis D103
Source: Microbiol Spectr. 2024 Nov 7;12(12):e01147-24. doi: 10.1128/spectrum.01147-24 (PMC11619478; doi:10.1128/spectrum.01147-24)

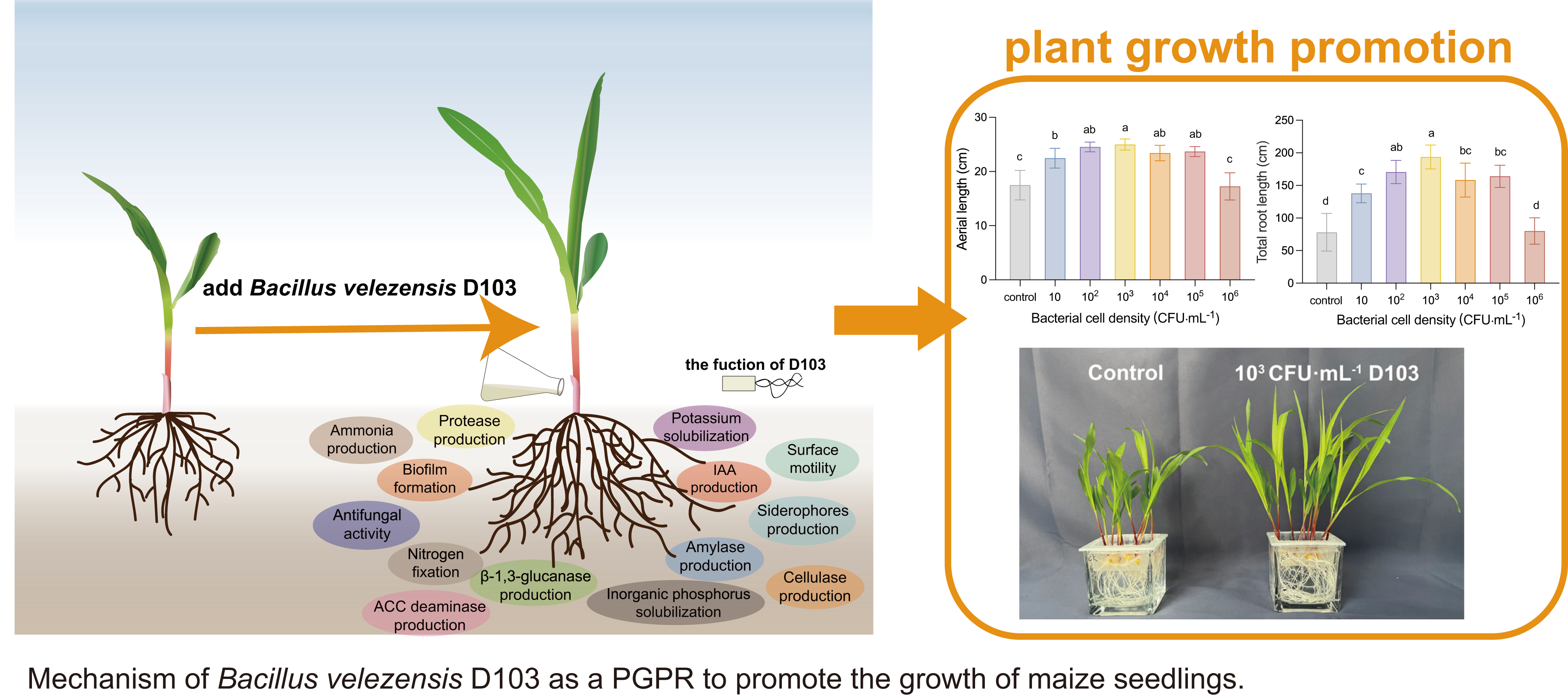

Supplement: Fig. S6 — Mechanism of Bacillus velezensis D103 as a PGPR to promote the growth of maize seedlings. [file spectrum.01147-24-s0002.tif]
